# Technical and procedural comparison of two different cryoballoon ablation systems in patients with atrial fibrillation

**DOI:** 10.1007/s10840-021-01035-6

**Published:** 2021-07-28

**Authors:** Sven Knecht, Christian Sticherling, Laurent Roten, Patrick Badertscher, Laurève Chollet, Thomas Küffer, Florian Spies, Antonio Madaffari, Aline Mühl, Samuel H. Baldinger, Helge Servatius, Stefan Osswald, Tobias Reichlin, Michael Kühne

**Affiliations:** 1 Cardiology/Electrophysiology, University of Basel Hospital, Petersgraben 4 4031, Basel, Switzerland; 2 Cardiology/Electrophysiology, Inselspital, University Hospital Bern, Bern, Switzerland

**Keywords:** Cryoballoon ablation, Atrial fibrillation, Technical specification

## Abstract

**Purpose:**

The aim was to report procedural and technical differences of a novel cryoballoon (NCB) ablation catheter for pulmonary vein isolation (PVI) compared to the standard cryoballoon (SCB) catheter.

**Methods:**

Consecutive patients with atrial fibrillation (AF) undergoing PVI using the NCB and the SCB were included. Procedural parameters, technical differences, acute efficacy, and safety are reported.

**Results:**

Eighty patients (age 66 ± 10 years, ejection fraction 57 ± 10%, left atrial volume index 40 ± 6 ml/m^2^) were studied. With the NCB, 156 of 158 PVs (99%) were isolated compared to isolation of 159 of 159 PVs (100%) with the SCB. The median number of freezes in the NCB and the SCB group was 6 (IQR 5–8) and 5 (IQR 4–7), respectively (*p* = 0.051), with 73% and 71% of the PVs isolated with a single freeze, respectively. Nadir temperature and temperature at isolation were − 59 ± 6 °C and − 45 ± 17 °C in the NCB group and − 46 ± 7 °C and − 32 ± 23 °C in the SCB group, respectively (both *p* < 0.001) with no difference in time to isolation (TTI). Procedural differences were observed for the total procedure time (84 ± 29 min in the NCB group and 65 ± 17 min in the SCB group, *p* = 0.003). There was a peri-procedural stroke in one patient in the NCB group. Differences in catheter design were observed that may account for the differences in temperature recordings and ice cap formation.

**Conclusions:**

Acute efficacy and TTI were similar with the NCB compared to the SCB. Measured temperatures were lower with the NCB, most likely due to differences in catheter design.

**Supplementary Information:**

The online version contains supplementary material available at 10.1007/s10840-021-01035-6.

## Introduction

Pulmonary vein (PV) isolation (PVI) using a cryoballoon (CB) ablation catheter was introduced more than 10 years ago and was shown to be non-inferior compared to point-by-point radiofrequency ablation in two randomized controlled trials [1–3]. Early CB ablation has been shown to result in fewer recurrences of atrial fibrillation (AF) than antiarrhythmic therapy in two randomized trials [4, 5]. Until recently, all procedural and outcome data were based on a single CB ablation system. Over the years, several device generations were released with advancements regarding cooling properties and real-time PV recordings [6, 7]. The most recently introduced version is the 4th generation Arctic Front Advance Pro (Medtronic, Minneapolis, MN, USA) hereinafter called the standard cryoballoon (SCB) [8, 9].

A novel cryoballoon (NCB) ablation catheter (POLARx, Boston Scientific, Marlborough, MA, USA) for PVI has recently been introduced. The catheter design of the NCB is different compared to the SCB, which has an impact on cooling properties, temperature measurements, and temperature behavior during CB ablation. Detailed data on technical differences, procedural details, acute efficacy, and safety of the NCB in particular are needed, after the market launch of the NCB. Safety data is particularly important since a transient ischemic attack with left-sided hemiparesis occurred in the first reported series of 57 patients with the NCB [10].

The purpose of this study was to report procedural parameters, technical differences, acute efficacy, and safety during an initial experience at two centers with the NCB compared to the SCB ablation catheter in patients undergoing catheter ablation of AF.

## Methods

### Study population

The non-randomized study population consisted of 80 consecutive patients undergoing CB ablation at two centers. The first 40 patients undergoing PVI using a standardized ablation protocol with the NCB (28-mm, short-tip POLARx, Boston Scientific) and 40 preceding patients using the SCB (28-mm Arctic Front Advance Pro, Medtronic, Minneapolis, MN, USA) were included at two centers. Exclusion criteria were the presence of long-standing persistent AF and a history of a previous left atrial procedure for PVI. Intracardiac thrombi were ruled out by transesophageal echocardiography before the procedure. All patients underwent pre-procedural imaging, either by computed tomography or by cardiac magnetic resonance imaging. Written informed consent was provided by all patients prior to the procedure. The study was approved by the local ethics committee on human research and complied with the Declaration of Helsinki.

### Cryoballoon ablation using the standard cryoballoon

The ablation procedure was performed under conscious sedation using midazolam, fentanyl, and propofol. Vascular access was obtained via the right femoral vein. A decapolar deflectable catheter (EZ STEER, Biosense Webster, Diamond Bar, CA, USA; or Dynamic XT, Boston Scientific) was positioned in the right subclavian vein for pacing of the phrenic nerve during ablation of all right superior PVs. Phrenic nerve capture was confirmed by continuous palpation and/or monitoring of the compound motor action potential on the surface ECG. Transseptal puncture was performed under fluoroscopic guidance. The activated clotting time was kept at a target of 350 s using intravenous heparin. The intracardiac electrograms and surface electrograms were displayed on an oscilloscope and recorded at a speed of 100 mm/s (Sensis, Siemens, Erlangen, Germany).

In the SCB group, the steerable 12F inner (15F outer) diameter sheath (FlexCath Advance, Medtronic, MN, USA) was positioned in the left atrium after transseptal puncture and was continuously flushed with heparinized saline. The sheath has a radiopaque marker located 5 mm proximal to the sheath tip. Only the 28-mm SCB (Arctic Front Advance Pro, Medtronic, 10.5F shaft diameter, 8-mm tip length) was used in this study. The catheter handle has a deflection mechanism and a blue push button for balloon elongation for re-sheathing. The SCB is used in conjunction with a console (CryoConsole, Medtronic) providing the nitrous oxide (N_2_O) from a tank. The N_2_O is injected into the balloon via 8 injection jets.^9^

A 20-mm octapolar inner lumen spiral mapping catheter (Achieve, Medtronic, Minneapolis, MN, USA) was used in order to visualize PV signals. PVs were isolated in the following sequence: left superior, left inferior, right inferior, and right superior.

After obtaining PV occlusion by optimal alignment of the sheath, the catheter, and the PV, freezing cycles with a standard duration of 180–240 s were started. Target temperatures were − 40 °C and/or PV isolation (time to isolation) within 60 s. Freezing cycles were prematurely terminated when − 60 °C was reached or in case of phrenic nerve palsy. The endpoint of the ablation was the elimination of all PV potentials on the spiral mapping catheter. No “bonus” freezes were applied. During the thawing phase, the blue push button was advanced to elongate the balloon before + 20 °C was reached. At that temperature, the balloon deflates automatically.

### Procedural differences with the novel cryoballoon

The NCB is used with the SMART FREEZE (Boston Scientific) console providing the N_2_O. The design of the NCB ablation system with a double-balloon layer, an internal balloon thermocouple at the shaft, and N_2_O delivery is very similar to that of the SCB. The pressure within the NCB remains stable from inflation to ablation, and the fluid flow during ablation is 7800 standard cubic centimeter per minute (sccm) compared to 7200 sccm for the SCB [10].

The sheath (POLARSHEATH, Boston Scientific) is a 12.7 French inner (15.9F outer) diameter deflectable sheath with a radiopaque marker 2.5 mm proximal to the sheath tip and a 155° angle of distal deflection. The distal end of the sheath appears softer to manual palpation compared to the NCB. The sheath is delivered without a stopcock.

The POLARx NCB catheter has a shaft diameter of 11.8 F and is currently available with a balloon diameter of 28 mm and with a long (12 mm) and a short (5 mm) catheter tip. For the purpose of this comparison, only the short-tip version of the NCB was used. The handle of the NCB has a steering lever with a tension nob and a slider switch (for manual deflation (only if >  + 20 °C) and extension of the balloon for re-sheathing) which shows procedure status with an LED color code (green: ready; blue: inflation/thaw; flashing blue: ablation). No specific target temperatures were used because of limited available data, but reaching TTI < 60 s was attempted. Applications were prematurely terminated before freezing cycles are automatically terminated at a temperature of − 70 °C. Identical to the SCB, the balloon deflates automatically at a temperature of + 20 °C. Real-time signals were recorded with a 20-mm loop diameter octapolar spiral mapping catheter (POLARMAP, Boston Scientific).

### Technical ex vivo characterization

In order to understand potential differences in cooling properties and temperature recording, the NCB and SCB were dissected (removal of the balloon) to describe and measure their technical specifications with focus on the position of the injection coil, the thermocouple, and the backflow of the N_2_O gas.

For semi-quantitative assessment of the ice cap formation as a surrogate of the heat transfer from the surrounding to the CB, three freezing cycles for three catheters in both groups were performed in a static water bath with 37 °C [7]. Pictures of the ice cap formation after 60, 120, 180, and 240 s in two perpendicular views were taken and the boundaries of the ice caps were delineated. The minimal and maximal ice coverage was used to characterize the homogeneity of their freezing capabilities over time.

### Post-ablation management

Oral anticoagulation was continued for at least 2 months. All antiarrhythmic drugs were stopped after the procedure. This was an acute study and follow-up ended at hospital discharge.

### Outcome measures

Technical differences, procedural parameters, failure rate (defined as catheter malfunction requiring a switch to a second CB catheter), acute efficacy, and safety are reported.

Acute efficacy was defined as PVI on a per patient and per PV basis using the CB. The number of freezing cycles per patient and per PV and the percentage of PVs which could be isolated with a single application (“single-shot isolation”) was determined. With regard to freezing properties, we report nadir temperatures, temperature at isolation of the PV and time to isolation (TTI), and the percentage of recorded real-time signals. In addition, procedure time, LA dwell time, net ablation time, and fluoroscopy time are reported.

Reported complications are limited to peri-procedural complications and post-procedural complications occurring before hospital discharge.

### Statistical analysis

Continuous variables are presented as mean ± one standard deviation or as median and interquartile range (IQR) in case of skewed distribution. For continuous variables, comparisons were made using Student’s *T*-test, or Mann–Whitney *U* test, as appropriate. Discrete variables were compared using Fisher’s exact test. A *p*-value < 0.05 was considered to indicate statistical significance. Analysis was performed using SPSS (IBM SPSS Statistics, Version 23.0, Armonk, NY, USA).

## Results

### Baseline characteristics

The study population consisted of 80 patients referred for PVI (65% male, age 66 ± 10 years). The mean left ventricular ejection fraction was 57 ± 10%, left atrial size (parasternal long axis view) was 40 ± 6 mm, and indexed left atrial volume (LAVI) was 39 ± 13 ml/m^2^. Left common PVs were present in two patients in the NCB group and one in the SCB group. Baseline characteristics are shown in Table 1. There were no differences between the NCB group and the SCB group with regard to baseline characteristics.

**Table 1:** Patient characteristics

	Standard CB (*n* = 40)	Novel CB (*n* = 40)	*p*-value
Age (years)	66 ± 9	65 ± 11	0.675
Men	26 (65)	26 (65)	1.000
Paroxysmal AF	28 (70)	23 (58)	0.352
BMI (kg/m^2^)	27 ± 4	28 ± 7	0.897
Left atrial size (mm)	40 ± 6	40 ± 6	0.973
Left atrial volume index (ml/m^2^)	41 ± 13	36 ± 12	0.188
Left ventricular ejection fraction (%)	58 ± 7	57 ± 12	0.899
Hypertension	20 (50)	20 (50)	1.000
CAD	7 (18)	11 (28)	0.422
Heart failure	1 (3)	5 (13)	0.201

Continuous variables are shown mean ± standard deviation and categorical parameters as numbers and percentage

*AF*, atrial fibrillation; *BMI*, body mass index; *IQR*, interquartile range; *CAD*, coronary artery disease

### Technical and ex vivo characterization

**Fig. 1:**
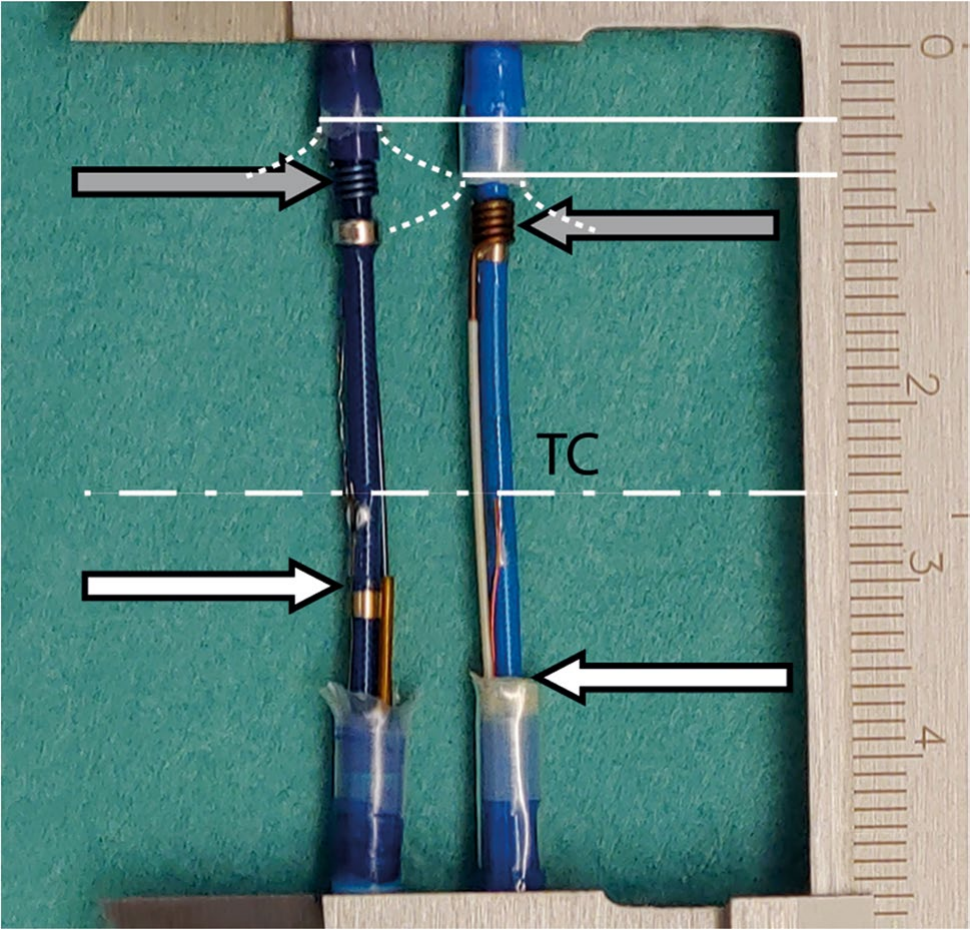
Dissected NCB (left) and SCB catheter (right). The white lines represent the position of the pole of the balloon and the dash-dotted line highlights the position of the thermocouple (TC). The gray arrows represent the position of the injection coils and the white arrows the position of the gas outflow

Removal of the balloon exposes the distal tip of the catheters which contain the thermocouple and the injection coil (Fig. 1). The middle of the injection coil (Fig. 1, gray arrow) is 2.5 mm and 3.5 mm away from the distal dip of the CB in the NCB and the SCB, respectively. The thermocouple (TC) is located at 25 mm from the tip for both catheter designs (dash-dotted line). Backflow of the gaseous N_2_O from the balloon inner lumen is realized over a tube 30 mm proximal from the tip for the NCB and the catheter shaft inner lumen at a distance of 35 mm proximal of the tip for the SCB. This results in a distance between the TC and the outflow of the cold gas of 5 mm for the NCB and 10 mm for the SCB. A comprehensive summary of the technical specifications can be found in Table 2.

**Table 2:** Technical specifications

	Standard CB	Novel CB
General specifications		
Shaft diameter (F)	10.5	11.8
Balloon design	Double-layer	Double-layer
Balloon size (mm)	23 & 28	28
Tip length (mm)	8	5 (short tip) or 12 (long tip)
Handle	Slider switch and push button for balloon elongation	Tension nob and a slider switch
Handle procedure status	None	LED color code Green: ready Blue: inflation/thaw Flashing blue: ablation
N_2_O injection	8-hole coil	8-hole coil
N_2_O fluid flow during freeze (sccm)	7200 (28 mm)	7800
Pressure during freeze (psi)	530–600	< 525, constant after approx. 12 s
Automatic termination	None	Temperature < − 70 °C
Sheath outer diameter (F)	15.0	15.9
Sheath stopcock	yes	no
Measured specifications		
Location of injection coil (mm)	3.5 (from pole of balloon)	2.5 (from pole of balloon)
Location of TC (mm)	25 (from tip)	25 (from tip)
	15 (from coil)	18 (from coil)
Location of gas outflow (mm)	10 (proximal of TC)	5 (proximal of TC)

*CB*, cryoballoon; *TC*, thermocouple

**Fig. 2:**
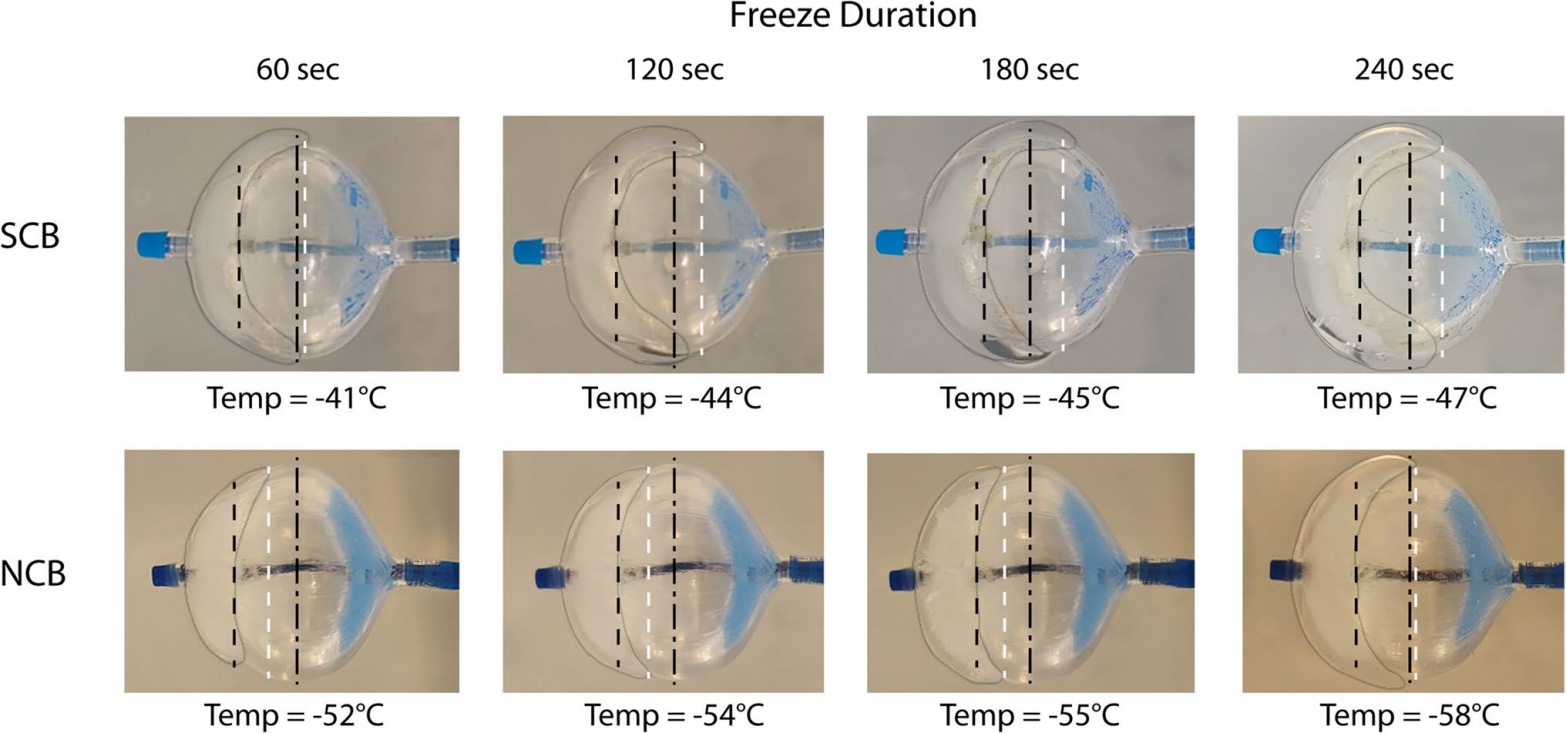
Exemplary temporal evolution of the ice cap formation (border highlighted) for the SCB (top row) and NCB (bottom row) at 60, 120, 180, and 240 s. The black dash-dotted line represents the middle of the CB (equator), the black dashed line represents the minimal coverage, and the white dashed line represents the maximal coverage. Note the slower increase of the maximal coverage with the NCB

Nadir CB temperatures of the freezing cycles in the water bath were similar to the temperature in the *in vivo* analyses (− 59 °C for the NCB and − 47 °C for the SCB). Analysis of the ice cap formation as a surrogate for effective heat transfer to the balloon revealed an inhomogeneous cooling of the distal hemisphere of the CBs (Fig. 2) for both CB types and over the entire freezing cycle. Local ice formation to or beyond the equator of the balloon to the proximal hemisphere could be observed for 9 of 9 freezes (100%) for the SCB and 6 of 9 freezes (67%) for the NCB after 180 s. Whereas maximal coverage increased over the duration of the freeze (Fig. 2, white dashed line) with a later increase in coverage for the NCB, the minimal coverage did not evolve (Fig. 2, black dashed line). A movie of the head-to-head comparison of the ice cap formation (8 times normal speed) can be found in the Supplementary information (Supplementary movie).

### Procedural data

The procedural endpoint of PVI was reached in 38 of 40 patients (95%) with the NCB compared to 40/40 (100%) with the SCB (*p* = 0.494). On a per vein basis, 156 of 158 PVs (99%) were isolated with the NCB compared to isolation of 159 of 159 PVs (100%) with the SCB (*p* = 0.248). There was one right superior PV which could not be isolated despite 4 attempts with the NCB and one right inferior PV that could not be reached despite changing to a stiff guidewire instead of the spiral mapping catheter for support in combination with the NCB. No touch-up radiofrequency ablations were performed.

The median total number of freezes in the NCB and the SCB group was 6 (IQR CI 5–8) and 5 (IQR CI 4–7), respectively (*p* = 0.051). A single-shot isolation was achieved in 116 of 158 PVs (73%) with the NCB compared to 113 of 159 PVs (71%) in the SCB group (*p* = 0.707).

Across all PVs, mean nadir temperature was − 59 ± 6 °C in the NCB group and − 46 ± 7 °C in the SCB group (*p* < 0.001). With a value of − 45 ± 17 °C in the NCB group, the temperature at isolation across all PVs was significantly lower compared to − 32 ± 23 °C in the SCB group (*p* < 0.001). TTI could be determined in 97 of 159 (61%) PVs with NCB compared to 92 of 158 (58%) PVs with SCB (*p* = 0.649), and TTI was similar with 52 ± 33 s in the NCB group and 50 ± 37 s in the SCB group (*p* = 0.735). These findings were consistent in the different PVs. Nadir temperature, temperature at isolation, and TTI for individual PVs are given in Table 3.

**Table 3:** Procedural data

	Standard CB (*n* = 40)	Novel CB (*n* = 40)	*p*-value
Procedure time (min)	65 ± 17 (62)	84 ± 29 (84)	0.003
LA dwell time (min)	47 ± 22 (43)	57 ± 25 (54)	0.053
Net ablation time (s)	1064 ± 296 (955)	1086 ± 369 (975)	0.832
Fluoroscopy time (min)	20 ± 7 (15)	25 ± 36 (18)	0.083
Single-shot isolation LSPV	31 (78)	33 (83)	0.781
Nadir temperature LSPV (°C)	− 48 ± 7 (47)	− 61 ± 6 (− 61)	< 0.001
Temperature at isolation LSPV (°C)	− 35 ± 10 (− 37)	− 50 ± 10 (− 50)	< 0.001
Time to isolation LSPV (s)	45 ± 23 (37)	53 ± 25 (45)	0.131
Single-shot isolation LIPV	27 (68)	32 (80)	0.310
Nadir temperature LIPV (°C)	− 44 ± 5 (− 45)	− 56 ± 5 (− 56)	< 0.001
Temperature at isolation LIPV (°C)	− 23 ± 38 (− 35)	− 43 ± 22 (− 47)	< 0.001
Time to isolation LIPV (s)	47 ± 42 (43)	55 ± 34 (40)	0.561
Single-shot isolation RSPV	30 (75)	25 (63)	0.335
Nadir temperature RSPV (°C)	− 47 ± 6 (− 47)	− 60 ± 7 (− 61)	< 0.001
Temperature at isolation RSPV (°C)	− 34 ± 8 (− 35)	− 41 ± 16 (− 43)	0.028
Time to isolation RSPV (s)	52 ± 42 (37)	45 ± 37 (30)	0.506
Single-shot isolation RIPV	25 (63)	26 (65)	0.815
Nadir temperature RIPV (°C)	− 47 ± 5 (− 47)	− 59 ± 6 (− 58)	< 0.001
Temperature at isolation RIPV (°C)	− 37 ± 8 (38)	− 46 ± 8 (− 49)	< 0.001
Time to isolation RIPV (s)	62 ± 44 (45)	55 ± 37 (37)	0.647
Interrupted freezing cycles	1.3 ± 1.5 (1)	1.2 ± 1.1 (1)	0.551

Continuous variables are shown mean ± standard deviation (median) and categorical parameters as numbers and percentage

*IQR*, interquartile range; *LSPV*, left superior pulmonary vein; *LIPV*, left inferior pulmonary vein; *RSPV*, right superior pulmonary vein; *RIPV*, right inferior pulmonary vein; *LA*, left atrial

The total procedure time was different between the groups (Table 3). After the exclusion of the first 20 cases of the NCB group to account for a training effect, the difference between procedure duration (SCB 65 ± 17 and NCB 84 ± 29, *p* = 0.026) remained significant.

There were no technical failures with the SCB, and there were four technical failures (10%) requiring a second NCB: A “blood detection error” occurred in two cases, and there was one case with a “pressure too high” alert (persistent despite changing cable), all resulting in a catheter exchange. There was one malfunction associated with the slider switch with impaired elongation of the distal catheter tip resulting in bending of the catheter tip during balloon inflation. This impaired catheter maneuverability so it had to be exchanged. Interruption of a freeze due to low temperatures (< − 70 °C for the NCB and <  − 60 °C for the SCB) were observed in 9 patients (23%) for the NCB and in 4 patients (10%) for the SCB (*p* = 0.225).

### Complications

There were no peri-procedural complications in the SCB group, and there was a peri-procedural stroke due to air embolism and transient phrenic nerve palsy in one patient in the NCB group. Whereas the phrenic nerve palsy was only transient during the procedure, the air embolism caused an initial left-sided hemiparesis with progression to coma 6 h after the procedure. In this patient, air could be aspirated via the POLARSHEATH after the exchange of the SL1 sheath used for transseptal puncture. Fortunately, the patient recovered with only minimal symptoms after 48 h of coma. The patient was discharged from the hospital 7 days after the procedure.

## Discussion

The main findings of this study are (1) PVI with a “single-shot isolation” rate of approximately 72% can be performed with a slightly but significantly longer procedure time and a similar net ablation time and fluoroscopy duration using the NCB compared to the SCB. (2) Ablation with the NCB is associated with markedly lower temperature recordings compared to the SCB, however, with a similar TTI between the two groups, suggesting a similar biological effect. (3) There are small but noticeable differences in catheter design between the NCB and the SCB that may account for differential temperature recordings. (4) The freezing pattern as assessed by the ice cap formation is inhomogeneous in both catheters with consistent coverage of the distal hemisphere up to the balloon equator and beyond only with the SCB. (5) With the occurrence of one stroke caused by air embolism, further studies are needed to document safety with the NCB.

CB ablation is an established tool to achieve PVI and has been used for > 15 years [11]. Different generations of catheters from the same manufacturer with enhanced cooling and PV signal recording capacities were released over time [6]. A recent report from the largest single-center experience (> 1000 ablations) using the SCB showed evidence of a persistent learning curve even in experienced operators [12]. Therefore, the introduction of a novel device such as the NCB, albeit very similar in design, warrants cautious use in the early clinical phase and accurate comparisons to available data with the SCB.

The fact that PVI can be successfully achieved with the NCB is reassuring from an efficacy standpoint. Currently, we can only report data on acute efficacy for the NCB. However, the similar time required to isolate a PV (reported as the TTI) measured with the NCB compared to the SCB suggests a similar biological effect. In addition, the quantitative evaluation of the size of the ice cap on the distal hemisphere of the balloon showed small differences between the two groups. With the duration of the freezing cycle, we observed a slight increase of the minimal area of the ice cap coverage up to 120 s only. After that, only local areas increased to the equatorial area of the balloon, with later coverage for the NCB. As shown before with the 2nd generation CB, the SCB consistently revealed freezing capabilities locally beyond the equator of the balloon after 180 s whereas this could only be observed in two-thirds of freezing cycles with the NCB in this study [7]. Homogeneous cooling with consistent ice cap formation over the entire distal hemisphere of the balloon was not observed for any freezing duration with either balloon type. This should be kept in mind in addition to obtaining occlusion when aligning the CB at the ostium of the PVs for both catheters. To address the issue of inhomogeneous freezing capabilities, balloon rotation might be advisable for unsuccessful applications despite perfect occlusion, but this was not tested in this study.

The TTI has been identified as a predictor of persistent electrical isolation [13, 14]. In our study, TTI was found to be similar in the two groups despite the markedly lower recorded measured temperatures (lower nadir temperature and temperature at isolation) with the NCB. In the first report on acute success of the NCB, the authors describe the constant pressure inside the NCB as the main difference compared to that in the SCB where pressure increases inside the balloon during ablation with full valve opening [10]. On close inspection of the cooling technology of the catheter after removal of the two balloon layers, we identified differences in catheter design between the two ablation systems that are most likely the cause for the lower recorded temperatures with the NCB. Specifically, the position and injection orientation of the N_2_O injection coil, the different N_2_O flow, the difference between gaseous backflow relative to the TC, or a combination of these different factors might mainly account for these differences in temperature measurements. Additionally, the higher compliance of the NCB could result *in vivo* in a movement of the thermocouple towards the distal tip where the main source of the cooling is, thereby resulting in lower temperature measurements. However, we did not observe a higher degree of balloon deformation when positioning the balloon at the PV ostium with the NCB compared to that with the SCB. Finally, a difference in the insulating capabilities of the double-layer CB material might also play a role, but this was not assessed in this study.

Safety is of utmost importance when introducing a novel technology, and CB ablation has been shown to be associated with a low peri-procedural complication rate based on data with the SCB [4, 5, 15, 16]. Both in our series and the first report on ablation using the NCB, there was one stroke or transient ischemic attack. Based on these early data, no definitive conclusions on the safety of the NCB can be drawn [10].

### Limitations

This is a small study from two centers analyzing a novel device with an already approved technology for PVI in a non-randomized fashion. Therefore, an unaccounted confounding variable may be present. Furthermore, the findings may be impacted by a learning curve as the data stem from an initial series using NCB from experienced SCB operators. Finally, this is an acute study with no follow-up information after hospital discharge. Whether the reported findings impact chronic success rates warrants further investigation.

In the *in vitro* test setup with the quantification of the ice cap formation, we delineate the temperature boundary at 0 °C. This allows a direct comparison of the two catheter types; however, for irreversible cell destruction, tissue temperatures of − 40 °C are required. In consequence, the effective cooling area of the CB on the distal hemisphere might be smaller.

## Conclusions

Acute efficacy and TTI were similar with the NCB compared to those with the SCB despite lower recorded temperatures at the time of isolation and lower nadir temperatures. This may be explained by small but significant differences in catheter design. Future studies will need to determine whether these differences impact success rates in the long term and to document safety.

## Supplementary Information

Below is the link to the electronic supplementary material.

Movie file showing parallel a 240 sec freeze in 8 times normal speed for both catheters in a water bath. (MP4 31386 KB)
